# Clinical Outcomes of 217 Patients with Acute Erythroleukemia According to Treatment Type and Line: A Retrospective Multinational Study

**DOI:** 10.3390/ijms18040837

**Published:** 2017-04-14

**Authors:** Antonio M. Almeida, Thomas Prebet, Raphael Itzykson, Fernando Ramos, Haifa Al-Ali, Jamile Shammo, Ricardo Pinto, Luca Maurillo, Jaime Wetzel, Pellegrino Musto, Arjan A. Van De Loosdrecht, Maria Joao Costa, Susana Esteves, Sonja Burgstaller, Reinhard Stauder, Eva M. Autzinger, Alois Lang, Peter Krippl, Dietmar Geissler, Jose Francisco Falantes, Carmen Pedro, Joan Bargay, Guillermo Deben, Ana Garrido, Santiago Bonanad, Maria Diez-Campelo, Sylvain Thepot, Lionel Ades, Wolfgang R. Sperr, Peter Valent, Pierre Fenaux, Mikkael A. Sekeres, Richard Greil, Lisa Pleyer

**Affiliations:** 1 Instituto Português de Oncologia de Lisboa (IPOL), 1200-795 Lisbon, Portugal; sesteves@ipolisboa.min-saude.pt; 2 Institut Paoli Calmettes, Marseille, France and Yale New Haven Hospital, New Haven, CT 06512, USA; thomas.prebet@yale.edu; 3 Hopital Saint-Louis, Assistance Publique-Hôpitaux de Paris (AP-HP), Paris Diderot University, 75010 Paris, France; itzykson@gmail.com (R.I.); lionel.ades@sls.aphp.fr (L.A.); pierre.fenaux@sls.aphp.fr (P.F.); 4 Hospital Universitario de Leon, 24071 Leon, Spain; mail@fernandoramosmd.es; 5 University Hospital of Halle, 06120 Halle, Germany; alah@medizin.uni-leipzig.de; 6 Rush University Medical Center, Chicago, IN 60612, USA; Jamile_Shammo@rush.edu; 7 Hospital Sao Joao, 4200-319 Porto, Portugal; rjsmpinto@gmail.com; 8 University Tor Vergata, 00173 Rome, Italy; luca.maurillo@uniroma2.it; 9 Cleveland Clinic Taussig Cancer Institute, Cleveland, OH 44195, USA; FENSTEJ@ccf.org (J.W.); SEKEREM@ccf.org (M.A.S.); 10 RCCS-CROB, Referral Cancer Center of Basilicata, 85028 Rionero in Vulture (Pz), Italy; p.musto@tin.it; 11 Department of Hematology VU University Medical Center, 1081 HV Amsterdam, The Netherlands; a.vandeloosdrecht@vumc.nl; 12 Centro Hospitalar Lisboa Norte Hospital Santa Maria, 1649-035 Lisbon, Portugal; mjoaocosta@gmail.com; 13 Department of Internal Medicine IV, Hospital Wels-Grieskirchen, 4600 Wels, Austria; sonja.burgstaller@klinikum-wegr.at; 14 Department of Internal Medicine V (Haematology and Oncology), Innsbruck Medical University, 6020 Innsbruck, Austria; reinhard.stauder@i-med.ac.at; 15 1st Department of Internal Medicine, Center for Oncology and Hematology, Wilhelminenspital, 1160 Vienna, Austria; eva-maria.autzinger@wienkav.at; 16 Internal Medicine, Hospital Feldkirch,6800 Feldkirch, Austria; alois.lang@lkhf.at; 17 Department of Internal Medicine, Hospital Fürstenfeld, 8280 Fürstenfeld, Austria; peter.krippl@lkh-fuerstenfeld.at; 18 Department for Internal Medicine, Klinikum Klagenfurt am Wörthersee, 9020 Pörtschach am Wörthersee, Austria; dietmar.geissler@kabeg.at; 19 Hospital Universitario Virgen del Rocio, 41013 Sevilla, Spain; jfalantes@gmail.com; 20 Hospital del Mar, 08003 Barcelona, Spain; MPedro@parcdesalutmar.cat; 21 Hospital Son Llatzer, 07198 Palma de Mallorca, Spain; jbargay@hsll.es; 22 Hospital Universitario, 15006 A Coruña, Spain; gdebari@canalejo.org; 23 Hospital de la Santa Creu i Sant Pau, 08026 Barcelona, Spain; AGarridoD@santpau.cat; 24 Hospital Universitario de la Ribera, 46600 Alzira, Spain; sbonanad@gmail.com; 25 Hospital Universitario de Salamanca, 37007 Salamanca, Spain; mdiezcampelo@usal.es; 26 Centre Hospitalier Universitaire, 49100 Angers, France; Sylvain.Thepot@chu-angers.fr; 27 Department of Internal Medicine I, Division of Hematology & Hemostaseology and Ludwig Boltzmann Cluster Oncology, Medical University of Vienna, 1090 Vienna, Austria; wolfgang.r.sperr@meduniwien.ac.at (W.R.S.); peter.valent@meduniwien.ac.at (P.V.); 28 3rd Med. Department, Paracelsus Medical University, 5020 Salzburg, Austria; r.greil@salk.at; 29 Salzburg Cancer Research Institute, 5020 Salzburg, Austria; 30 Cancer Cluster Salzburg, 5020 Salzburg, Austria; 31 Arbeitsgemeinschaft Medikamentöse Tumortherapie (AGMT), 5020 Salzburg, Austria

**Keywords:** acute erythroleukemia, azacitidine, decitabine

## Abstract

Acute erythroleukemia (AEL) is a rare disease typically associated with a poor prognosis. The median survival ranges between 3–9 months from initial diagnosis. Hypomethylating agents (HMAs) have been shown to prolong survival in patients with myelodysplastic syndromes (MDS) and AML, but there is limited data of their efficacy in AEL. We collected data from 210 AEL patients treated at 28 international sites. Overall survival (OS) and PFS were estimated using the Kaplan-Meier method and the log-rank test was used for subgroup comparisons. Survival between treatment groups was compared using the Cox proportional hazards regression model. Eighty-eight patients were treated with HMAs, 44 front line, and 122 with intensive chemotherapy (ICT). ICT led to a higher overall response rate (complete or partial) compared to first-line HMA (72% vs. 46.2%, respectively; *p* ≤ 0.001), but similar progression-free survival (8.0 vs. 9.4 months; *p* = 0.342). Overall survival was similar for ICT vs. HMAs (10.5 vs. 13.7 months; *p* = 0.564), but patients with high-risk cytogenetics treated with HMA first-line lived longer (7.5 for ICT vs. 13.3 months; *p* = 0.039). Our results support the therapeutic value of HMA in AEL.

## 1. Introduction

Acute erythroleukemia (AEL) is a rare subtype of acute myeloid leukemia (AML), accounting for 3–5% of all AML cases [1]. It is characterized by an expanded erythroid component with a variable, but increased, percentage of blasts [1]. Although recognized as a distinct entity by most classification systems, the diagnostic criteria have changed from system to system, which has been subject to discussion [2,3,4,5]. The recently-published WHO 2016 classification [6] advocates the use of blast percentage on the total cell population rather than that of the non-erythroid component. This reclassifies almost all cases of AEL into myelodysplasia (MDS) or AML subtypes [7,8].

Typical laboratory features include pancytopenia, few peripheral blood blasts, the presence of dysplasia in BM and peripheral blood, especially with dysplastic PAS-positive erythroblasts overexpression of the multidrug resistance (MDR) gene product P-glycoprotein, frequent occurrence of high-risk karyotypes, and a high frequency of mutations, especially of *TP53* [2,9,10,11,12,13,14]. In addition, AEL is frequently secondary to previous myelodysplastic syndrome (MDS) [15]. Consequently, it is associated with a poor prognosis, with a median overall survival (OS) of 3–14 months from diagnosis [1,2,10,14]. The only recurring molecular alteration reported has been translocation t(1;16) generating the fusion gene NFIA/CBFA2T3 [16]. Furthermore, a high proerythroblast/myeloblast ratio correlates with significant increases in cytogenetic aberrations, proliferation markers, and worse outcomes [1,17,18,19], although this is not consensual [10,14]. In fact, several authors believe that the association of AEL with adverse prognostic factors imparts the adverse prognosis, rather than the diagnosis of AEL itself [14,20].

Due to the rarity of the disease (2–5% of all leukemias), few publications focus on this entity alone, with single cases or case series predominating [9,10,15,21] and patients with AEL are usually treated similarly to patients with other types of AML [1,3]. When treated with intensive chemotherapy (ICT), the median OS of AEL patients range between 7.6 and nine months [14,22]. The poor results achieved with ICT in AEL are likely due to the adverse prognostic factors described above.

Hypomethylating agents (HMAs; azacitidine and decitabine) have become the first-line therapy of choice for patients with MDS [23,24], CMML [25,26,27], and AML [28,29,30,31,32,33,34,35,36] who are not candidates for, or decline, intensive chemotherapy (ICT) and/or allo-SCT. HMAs have demonstrated improved outcomes for patients with AML when compared to conventional care regimens, including ICT, low-dose cytarabine, or best supportive care (BSC) [28,29,30,37,38]. Despite some limitations, several studies indicate that the OS of older AML patients treated with HMAs may not be inferior to those treated with ICT [28,29,39,40,41,42,43,44].

The few existing studies of HMA in AEL report favourable response rates and survival times [21,45,46,47,48]. Larger patient series or randomized clinical trials are lacking. In this international effort, we report on the largest cohort of AEL patients in whom we describe baseline characteristics, overall response rates (ORR), and OS in those treated with HMAs or ICT. In an exploratory analysis, we also compare the treatment outcomes of patients receiving first-line HMA line with those treated with ICT.

## 2. Results

### 2.1. Total Acute Erythroleukemia (AEL) Cohort (n = 217)

The overall sample comprised 210 patients with AEL. Of these, 88 (41%) received treatment with HMA in the first or subsequent lines of therapy (82 were treated with azacitidine, six with decitabine) and 122 (56%) received ICT alone. Median age at diagnosis was 69 years (range: 28–88) for the HMA group, and 60 years (range: 20–86) for the ICT group. Poor cytogenetic risk was found in 51% of the HMA and 43% of the ICT groups. Baseline patient characteristics according to treatment group and line of therapy are detailed in Table 1. In the whole AEL cohort, 135 deaths were documented, 79 (59%) due to disease progression, 21 (15%) due to infection, 12 (9%) due to other causes, and in 23 cases (17%) the cause of death was unknown. The median follow-up of all patients was 7.7 (range, 0.2–148.5) months. One patient from the ICT group and four patients from the HMA group were not evaluable for PFS or OS (data regarding time to treatment start and/or death were missing) and, thus, were excluded from the survival analysis. For the total treated cohort (first-line HMA, second-line or later HMA, ICT), the median PFS was 7.1 (range: 6.3–9.4) months, the median OS was 11.1 (range: 9.8–14.3) months and the one-year survival rate was 49% (range: 42–57%) (Tables S1 and S2).

### 2.2. AEL Treated with HMA (n = 88)

In the cohort treated with HMAs, 41 patients (47%) received HMA as a front-line treatment, 45 as a second-line or later treatment, and two patients were excluded from the analysis as no data were provided regarding the treatment line of HMA. Prior disease-modifying treatments in patients receiving HMA as a second-line or later therapy included allo-SCT (5/45), ICT (40/45), low-dose cytarabine (5/45), and/or IMiDs (immunomodulatory agents, e.g., Lenalidomide) (4/45); four patients received concomitant growth factors, one patient received growth factors without prior disease-modifying treatment. The median time from initial diagnosis to treatment was 0.72 (range, 0.03–18.43) months in patients treated with first-line HMA, and 7.6 (range, 0.07–85.27) months in the group receiving HMA as a second-line or later treatment (*n* = 45). In patients treated with HMA, the median number of cycles in patients for whom data were available (*n* = 72) was five (range, 1–37); those treated with first-line HMA received a median of seven cycles (range, 1–37), and those treated in the second-line received a median of three cycles (range, 1–22). Those treated with azacitidine (*n* = 82) were treated with 28 day cycles: 35% received the schedule 5-2-2 (75 mg/m^2^ days 1–5, rest days 6–7, administer days 8–9), 32% received the schedule 1–7 (75 mg/m^2^ days 1–7), 26% received the schedule 1–5 (100 mg/m^2^ days 1–5), and 7% received other schedules. Those treated with decitabine (*n* = 6) received 15 mg/m^2^ for three days every six weeks. At the time of data assessment, 66 patients (76%) had died, of which seven died of subsequent allo-SCT complications. Twenty-two patients (24%) were alive; of these, nine had stopped treatment with HMA, nine were still on treatment with HMA (eight with azacitidine and one with decitabine), and four patients were alive at follow-up, but it was unknown whether they were still receiving HMA or not. The main reason for treatment discontinuation was disease progression (*n* = 39, 62%). Other reasons included infection/toxicity (*n* = 8, 12%), death (*n* = 8, 12%), allo-SCT (*n* = 5, 8%), and others (*n* = 4, 6%). Causes for death were similarly distributed between HMA and ICT treatment groups (Table S3).

Response data for patients treated with HMA were available for 75 patients. Among these, best overall response rate (ORR) according to the ELN criteria (complete, CR, or partial, PR) of patients treated with HMA was 40%; when including hematological improvement (HI), ORR rose to 59%; 27% had CR, 13% had PR, and 19% had HI; 35% of patients who were initially dependent on red blood cell transfusion achieved transfusion independence, and 29% of patients who were initially platelet transfusion dependent achieved transfusion independence (Table 2). Of those with an abnormal karyotype at the start of treatment, 11 (21%) of 51 HMA patients reached cytogenetic remission and 40 (53%) of 75 ICT-treated patients achieved cytogenetic remission. The median time to first response was 2.6 months (range, 0.6–27.4) and the median time to best response was 3.9 months (range, 0.66–38.3), respectively.

After a median follow-up of 12.3 (range, 0.03–35.2) and 4.8 (range, 0.0–68.8) months for patients treated with first-line HMA and second line or later HMA treatment, respectively, the median (range) PFS was longer for those treated with HMA in first-line treatment compared to second-line or later (9.4 (range 4.2–14.5) vs. 3.4 (2.0–6.3) months, respectively; Table 1). The median OS (range) was also longer for those treated with first-line HMA compared to second-line or later (13.7 (12.3–20.5) vs. 9.8 (4.6–13.5), respectively; Table S2).

**Figure 1:**
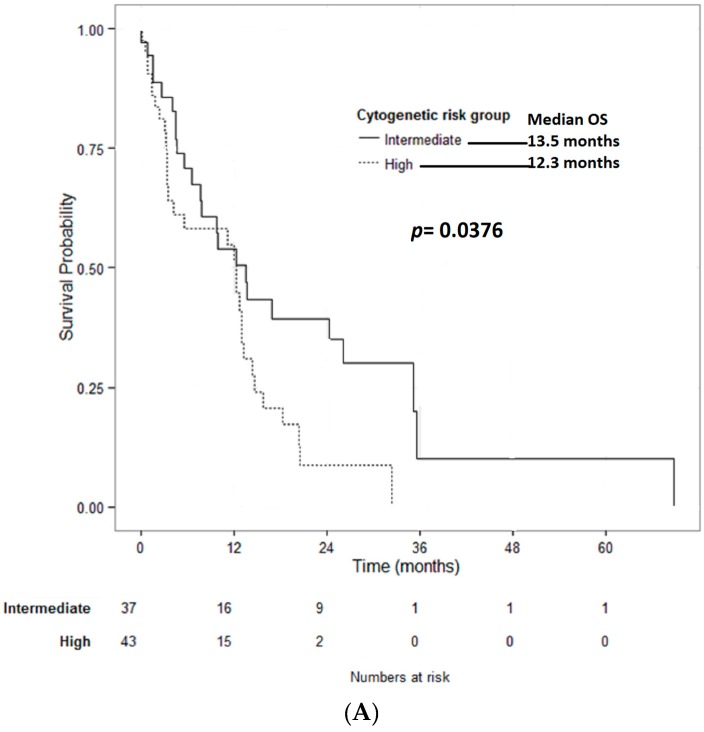

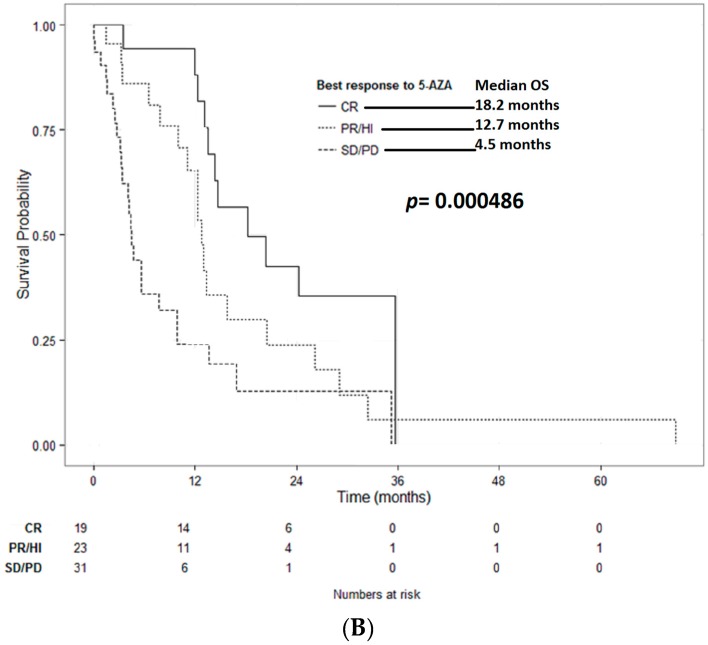
(**A**) Overall survival of HMA-treated patients stratified by cytogenetic risk group (total HMA cohort): the median OS for patients treated with HMA was superior for patients with intermediate-compared to high-risk cytogenetics (13.5 months vs. 12.3 months; *p* = 0.0376); and (**B**) the overall survival by response to HMA: the median survival in patients with CR was 18.2 months, 12.7 months in patients with PR or HI, and 4.5 months in patients with no response (SD or primary PD; *p* < 0.001).

The median OS for AEL patients treated with HMA (all treatment lines) was superior for patients with intermediate- compared to high-risk cytogenetics (13.5 vs. 12.3 months; *p* = 0.0376) (Figure 1A). AEL patients treated with first-line HMA with intermediate-risk cytogenetics had a median OS of 29.3, whereas those with high-risk cytogenetics had a median OS of 13.3 months (Table 3).

Ten (11.3%) patients had an allogeneic bone marrow transplant following treatment with HMA. The median OS in this subgroup was 9.66 months (range, 2.8–25).

In univariate analysis, response to HMA had a significant impact on OS (Figure 1B). The median survival in patients with CR was 18.2 months, 12.7 months in patients with PR or HI, and 4.5 months in patients with no response (stable disease, SD, or primary progressive disease, PD; *p* < 0.001).

### 2.3. AEL Treated with ICT (n = 122)

In the group of 122 patients receiving front-line ICT treatment, response data were available for 119 patients. The most frequently used (*n* = 81; 66%) induction regimen was Daunorubicin (45 or 60 mg/m^2^ × 3 days) with Cytarabine (100 mg/m^2^ bid × 7 days). Similar 3 + 7 regimens using Idarubicin 12 mg/m^2^ or Mitoxantrone 12 mg/m^2^ for three days, instead of Daunorubicin, were used in 25 (20%) and eight (7%) patients, respectively. Information regarding induction regimen was not available in eight (7%) patients.

ORR according to the ELN criteria was 72%; CR in 79 patients was 66%; PR in seven patients was 6%; SD in 16 patients was 13%; PPD in 17 patients was 14% (see Table 2). Data on HI was not assessed in this subgroup of patients, as this response form is considered irrelevant for AML-patients treated with ICT.

At the time of data assessment, 84 patients (69%) had died, and 37 (31%) were alive. The main cause of death was disease progression (65%) (Table S3).

Median follow-up was 7.8 (range, 0.03–148.5) months for patients treated with ICT. Median PFS was 8.0 months (range, 6.8–14.5) for AEL-patients treated with ICT (Table S1). Median OS for patients treated with ICT was 10.5 (range, 9.1–20.0) months (Table S2). Median OS for AEL-patients treated with ICT was not significantly superior for patients with intermediate- vs. high-risk cytogenetics (16.9 vs. 7.5 months; *p* = 0.277) (Table 3). For AEL-patients treated with ICT, the median OS of intermediate- vs. high-risk cytogenetics was 29.3 vs. 13.3 months, *p* = 0.0.039 (Table 3). In univariate analysis, the response to ICT had a significant impact on overall survival. The median OS in patients with CR was 23.17 months, as compared to 4.07 months in patients with PR, and 5.63 months in patients with no response (SD or primary PD; *p* < 0.001).

Twenty-three (18.8%) patients had an allogeneic bone marrow transplant following treatment with ICT. Median OS in this subgroup was 5.9 months (range, 2.0–17.9).

### 2.4. Comparison of AEL Treated with ICT vs. HMA

There were no significant differences in baseline characteristics (Table 1) or causes of death in the HMA vs. ICT group (Table 3).

AEL-patients treated with ICT had a higher rate of CR (66% vs. 30.8%; *p* < 0.001), and ORR according to the ELN criteria (CR + PR) (72% vs. 46.2%, *p* = 0.016) compared to patients treated with first-line HMA, respectively. Notably, there were significantly more progressions in the ICT group compared to the HMA group (14.3% vs. 7.7%, *p* = 0.004) and more disease stability in the HMA group (28.2% vs. 13.4%, *p* = 0.001).

Despite this higher response rate, there was no significant difference in median PFS (8.0 vs. 9.4 months; *p* = 0.342) or 1-year PFS rates (42% vs. 41%; *p* = 0.896) (Table S2). In multivariate analyses controlling for cytogenetic risk group and age, treatment with ICT was not superior to treatment with first-line HMA in prolonging PFS (*p* = 0.6907) (Table 4).

A likelihood ratio test was used to compare models with and without interaction between first line treatment and cytogenetic risk group: *p*-value = 0.0994.

**Figure 2:**
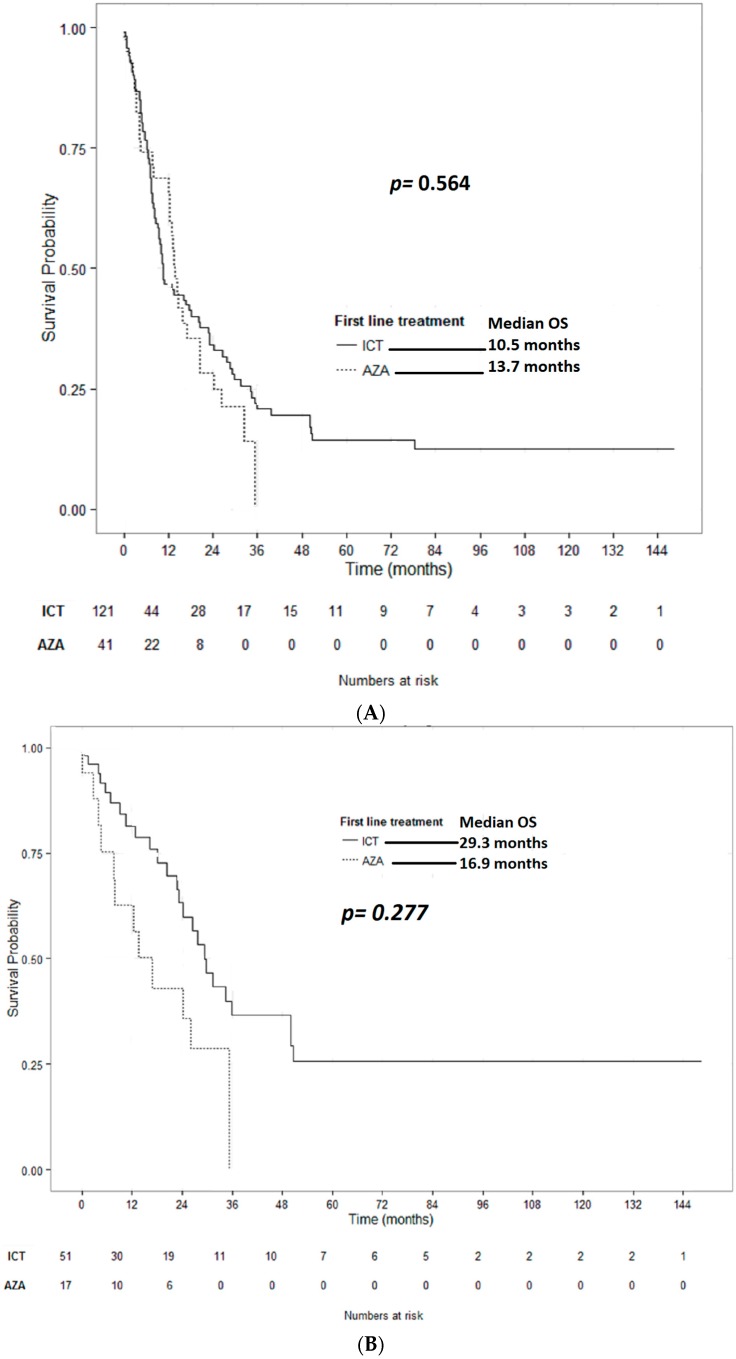

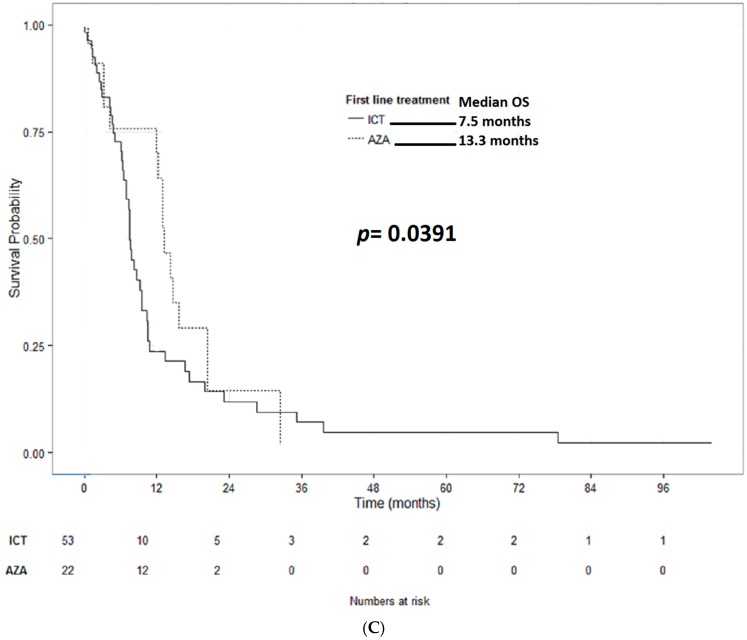
Overall survival of AEL patients stratified by type of first line treatment. (**A**) Total cohorts: median OS for patients treated with first-line HMA was similar to that of those treated with first-line ICT (13.7 months vs. 10.5 months; *p* = 0.564); (**B**) stratified by MRC intermediate cytogenetic risk: AEL-patients with intermediate-risk cytogenetics treated with first-line HMA did not have a significantly different median survival as compared to AEL-patients treated with first-line ICT (16.9 months vs. 29.3 months; *p* = 0.277); and (**C**) stratified by MRC high cytogenetic risk: AEL-patients with high-risk cytogenetics treated with first-line HMA had a significantly longer median survival as compared to AEL-patients treated with first-line ICT (13.3 months vs. 7.5 months; *p* = 0.0391).

Comparing AEL-patients treated with ICT vs. first-line HMA, no significant differences in 1-year survival rates (47% vs. 66%; *p* = 0.072) or median OS times could be detected (10.5 vs. 13.7 months; *p* = 0.564), respectively, though absolute numbers favored HMAs (Figure 2A). When stratified by the cytogenetic risk group, there was no significant difference in the median survival of AEL-patients with intermediate cytogenetic risk treated with ICT vs. first-line HMA (16.9 vs. 29.3 months; *p* = 0.277; Figure 2B). However, a shorter survival was detected for AEL-patients with high risk cytogenetics treated with ICT, as compared to those treated with first-line HMA (7.5 vs. 13.3 months; *p* = 0.039; Figure 2C). In multivariate analysis, controlling for age and cytogenetic risk, treatment with ICT was not superior to treatment with first-line HMA in prolonging OS (*p* = 0.2489), whereas both the MRC cytogenetic risk group (*p* < 0.0001) and age per additional year (*p* = 0.0032) did (Table 4).

## 3. Discussion

No prospective clinical trial has been conducted exclusively in patients with AEL. Little is known about the responses to specific drugs in AEL. Case reports and small series indicate possible efficacy of azacitidine [21,45,49], interferon-α [50], and even high dose erythropoietin combined with granulocyte colony-stimulating factor [51].

It was demonstrated several decades ago that HMA can induce erythroid differentiation and increase the synthesis of hemoglobin in both murine and human erythroleukemia cell lines in vitro [52,53,54]. In addition, the HMA decitabine was shown to induce down-regulation of the multidrug resistance (MDR) gene phospho-glycoprotein in a human erythroleukemia cell line, which coincided with modulation of response to cytostatic drugs [55,56].

We report here the largest series to date of patients with AEL treated with HMA. The overall response rate of 46% in the front-line setting, with a CR rate of 30% and an additional HI rate of 18%, in our cohort are encouraging and similar to those reported in other smaller studies [21,46,49]. Our study reinforces that, when treating AEL with HMA, any type of response, including hematological improvement, is beneficial. The observation that, despite a significantly lower ORR rate than ICT, PFS similarly suggests that the significantly higher SD rate also has an impact on survival. This highlights the importance of maintaining treatment in all patients who do not progress, even in the absence of marrow responses.

It is also noteworthy that initial responses were seen after a median of 79 days, but the best responses were documented after a median of 120 days, confirming that responses improve with continued treatment and reinforcing the importance of not interrupting treatment too early due to a lack of response.

When compared to AEL patients who were treated with ICT alone, those treated with HMA in as a first-line had similar progression-free and overall survival. This is significant considering the more advanced age of the HMA group. Older patients tolerate intensive chemotherapy poorly. In addition, aggressive treatment options are associated with long hospital admissions and poor quality of life, which may not be justified in an elderly patient group with a disease that is unlikely to be cured. HMA are administered in an outpatient setting and associated with reduced hospital admissions. Given the lack of a curative option for most patients and similar survival, the toxicity profile of HMA makes this option more attractive [57]. In addition, our data shows that adverse karyotype patients have better outcomes when treated with HMA compared to ICT. This suggests that HMA may be the preferred treatment option for older individuals with a poor prognosis karyotype, as is often seen in AEL.

Nevertheless, it is important to note that HMAs do not preclude the option of a bone marrow transplant. The therapeutic goal in younger patients with a donor should be to cure the disease and allo-SCT is the only option. Reduced intensity conditioning regimens have opened the option of allo-SCT to more elderly and frail patients but the toxicities associated with conventional intensive AML induction chemotherapy can increase the risk of death or compromise allo-SCT. It has been shown that Azacitidine before SCT does not significantly affect rates of remission, relapse, acute and chronic GVHD, and survival after transplant, and may actually be an alternative for inducing remission in patients with higher risk MDS [58], and eventually AEL [10]. Despite only 10 patients treated with HMA in our cohort having a subsequent SCT, their median survival is encouraging.

Our series analyses patients with AEL but we now know that there is great genetic heterogeneity in myeloid disorders, with a large variety of mutations having been described which have differing impacts on the natural history of the disease. Very recent analyses of the mutational profiles have significantly increased our understanding and prognostication of acute leukemias [59,60]. Future studies in this regard are needed in order to identify those patients with AEL who are most likely to respond to HMA.

## 4. Methods

### 4.1. Patient Population

Patient data were collected retrospectively and pooled from registries or patient files from 28 different Institutions representing eight different countries (Austria, France, Germany, Italy, Netherlands, Portugal, Spain, and USA). All subjects gave their informed consent for inclusion before they participated in the study. The study was conducted in accordance with the Declaration of Helsinki, and the protocol was approved by the Medical Ethics Committee of each individual centre. AEL diagnosis by WHO 2008 criteria was the only entry criterion and this was confirmed by local diagnostic laboratories. MRC cytogenetic risk stratification was applied to all patients.

Patients were included in the HMA group if they had received HMA at any stage of their treatment, whether first, or subsequent, lines. Patients in the ICT group must have been treated with ICT in first-line and never received HMA. Patients diagnosed between March 1998 and November 2014 were included. Treatment choice was made by the treating physician according to personal practice and local protocols. Seven AEL patients treated only with supportive care were proposed for the study, but their outcomes were not included in the analysis.

### 4.2. Definition of Endpoints

Response was defined according to European Leukemia Network (ELN) criteria for AML, and included complete remission (CR) and partial remission (PR) [61]. In addition, hematologic improvement (HI) was assessed according to the modified International Working Group (IWG) criteria 2006 [62]. OS was defined as time from start of treatment with HMA (either first- or second-line or later) or ICT to death from any cause, or last follow-up. Patients who underwent allogeneic stem cell transplantation (allo-SCT) after treatment with HMA or after ICT were censored at the date of allo-SCT. Progression-free survival (PFS) was defined as the time from the start of treatment until disease relapse/progression, or death from any cause.

### 4.3. Statistics

Descriptive statistics were used to describe the baseline patient characteristics. OS and PFS were estimated using the Kaplan-Meier method and the log-rank test was used for subgroup comparisons. The comparison of baseline features between the subgroups HMA and ICT was performed using the Pearson’s χ-squared test for categorical baseline variables and the Wilcoxon rank sum test for quantitative variables. In the exploratory analysis to evaluate the impact of treatment (HMA first-line vs. ICT) on OS and PFS we used the Cox proportional hazards regression model. Adjusted hazard ratios were calculated controlling for the potential confounding factors age and cytogenetic risk group. The likelihood ratio test was used to test the interaction between treatment and cytogenetic risk groups. All tests were two-tailed and *p*-values less than 0.05 were considered to be statistically significant. No adjustment was made for multiple comparisons. All analyses were performed using R [63].

## 5. Conclusions

Our data reinforces the utility of HMA in patients with acute erythroleukamia, especially those with poorest prognosis. Future studies in this regard are needed in order to identify those patients with AEL who are most likely to respond to HMA.

## Figures and Tables

**Table 1:** Baseline clinical and demographic characteristics according to treatment group and line.

Parameter	HMA All Lines (*n* = 88)	First-Line HMA (*n* = 41)	First-Line ICT (*n* = 122)	*p*-Value First-Line HMA vs. ICT
**Median age at diagnosis, years**	69	73	60	0.1698
(min–max)	(28–88)	(44–88)	(20–86)	
**Male gender, *n* (%)**	54 (61)	26 (63)	88 (72)	0.3919
**BM blasts at start of treatment**				
Median	22	22	24	0.8576
Mean (Standard Deviation)	25.8 (17.2)	25.8 (15.9)	27.1 (15.8)	
Unknown, *n* (%)	12 (14)	2 (5)	6 (5)	
**Hemoglobin at start of treatment, *n* (%)**				
≤10 g/dL	55 (63)	32 (78)	32/64 (50)	1.00
Pts. with unknown hemoglobin	3 (3)	0 (0)	71 (58)	
**Median WBC count at start of treatment, ×10^9^/L (min–max)**	2.35	2.42	1.81	0.7294
	(0.1–32.3)	(0.6–24.0)	(0.2–23.9)	
**Neutrophil count at start of treatment, *n* (%)**				
≤ 0.5 × 10^9^/L	34 (39)	18 (44)	18/57 (31)	0.7326
Pts. with unknown neutrophil count	5 (6)	1 (2)	79 (65)	
**Platelet count at start of treatment, *n* (%)**				
≤50 × 10^9^/L	54 (61)	24 (69)	62 (51)	0.8673
Unknown	3 (3)	0 (0)	10 (8)	
**AML subtype, *n* (%)**				
Primary	66 (75)	35 (85)	81 (66)	0.4373
Secondary	11 (13)	4 (10)	17 (14)	
Unknown	11 (13)	2 (5)	24 (20)	
**MRC cytogenetic risk group, *n* (%)**				
Good risk	1 (1)	0 (0)	0 (0)	0.6943
Intermediate risk	39 (44)	17 (42)	51 (42)	
Poor risk	45 (51)	22 (54)	53 (43)	
Unknown	3 (3)	2 (5)	18 (15)	

MRC = Medical research council.

**Table 2:** Responses of AEL patients treated with HMA or ICT.

	HMA All Lines (*n* = 75) ^1^	HMA 1st Line (*n* = 39) ^2^	HMA ≥ 2nd Line (*n* = 34) ^3^	ICT 1st Line (*n* = 119) ^4^
**Overall response acc. to ELN, *n* (%)**	30 (40.0)	18 (46.2)	10 (29.4)	86 (72.3)
Complete	20 (26.7)	12 (30.8)	7 (20.6)	79 (66.4)
Partial	10 (13.3)	6 (15.4)	3 (8.8)	7 (5.9)
**Overall response including HI, *n* (%)**	44 (58.7)	25 (64.1)	17 (50.0)	ND
**HI without marrow response**	14 (18.7)	7 (17.9)	7 (20.6)	ND
ANC	9 (12.0)	6 (15.4)	3 (8.8)	
RBC	7 (9.3)	5 (12.8)	2 (5.9)	
PLT	9 (12.0)	5 (12.8)	4 (11.8)	
**Transfusion independence, *n*/*n* (%) ^5^**				
RBC-TI	19/55 (35)	13/32 (40.6)	6/21 (28.6)	ND
PLT-TI	8/28 (29)	3/14 (21.4)	4/12 (33.3)	
**Stable disease**	26 (34.7)	11 (28.2)	15 (44.1)	16 (13.4)
**Primary disease progression**	5 (6.7)	3 (7.7)	2 (5.9)	17 (14.3)
**Time to first response, days ^6^**				ND
Median (min–max)	79 (18–822) ^7^	66 (18–233)	85 (30–822)	
**Time to best response, days ^8^**				
Median (min–max)	120 (20–1150) ^7^	143 (20–353)	89.5 (30–1150)	ND

^1^ Data available for 75 patients; ^2^ Data available for 39 patients; ^3^ Data available for 34 patients; ^4^ Data on HI was not assessed in this subgroup of patients, as this response form is considered irrelevant for AML-patients treated with ICT; ^5^ Evaluated in the subset of patients who were transfusion dependent at the start of HMA therapy; ^6^ Data available for 51 patients; ^7^ The longest time (822 days to fist response and 1150 days to best response) is a single patient. Other late responders are all ~200 days (6.6 months); and ^8^ Data available for 52 patients; and ND: not detected.

**Table 3:** Comparison of AEL patient characteristics and outcomes according to front-line treatment with ICT or HMA in univariate analysis.

Outcomes	First-Line ICT	First-Line HMA	*p*-Value
Overall response acc. to ELN, %	72.3	46.2	0.016
Complete response	64.4	30.8	<0.001
Partial response	5.9	15.4	0.101
Stable disease, %	13.4	28.2	0.001
Primary disease progression, %	14.3	7.7	0.004
Median time to best response, months	NA ^1^	89.5	NA ^1^
Median PFS, months	8.0	9.4	0.107
MRC intermediate cytogenetic risk	22.7	5.9	0.004
MRC high cytogenetic risk	6.5	11.3	0.279
1-year PFS, %	41.8	40.6	0.896
Median OS total cohort, months	10.5	13.7	0.564
MRC intermediate cytogenetic risk	16.9	29.3	0.277
MRC high cytogenetic risk	7.5	13.3	0.039
1-year OS total cohort, %	46.7	65.8	0.072

^1^ NA = not available.

**Table 4:** PFS and OS comparison for first-line treatment with HMA vs. ICT, controlling for cytogenetic risk group and age.

PFS Comparison	Hazard Ratio	95% CI	*p*-Value
First line AZA vs. ICT	0.90	0.54–1.51	0.6907
Cytogenetic risk group:			
High vs. Intermediate	1.86	1.19–2.90	0.0064
Age			
Per additional year	1.03	1.01–1.05	0.0118
**OS Comparison**	**Hazard Ratio**	**95% CI**	***p*-Value**
First line AZA vs. ICT	0.75	0.45–1.23	0.2489
Cytogenetic risk group			
High vs. Intermediate	2.40	1.54–3.69	<0.0001
Age			
Per additional year	1.03	1.01–1.05	0.0032

## Supplementary Materials

Supplementary materials can be found at www.mdpi.com/1422-0067/18/4/837/s1.

## References

[1] Santos F.P., Bueso-Ramos C.E., Ravandi F. (2010). Acute erythroleukemia: Diagnosis and management. Expert Rev. Hematol..

[2] Liu W., Hasserjian R.P., Hu Y., Zhang L., Miranda R.N., Medeiros L.J., Wang S.A. (2011). Pure erythroid leukemia: A reassessment of the entity using the 2008 World Health Organization classification. Mod. Pathol..

[3] Zuo Z., Polski J.M., Kasyan A., Medeiros L.J. (2010). Acute erythroid leukemia. Arch. Pathol. Lab. Med..

[4] Kasyan A., Medeiros L.J., Zuo Z., Santos F.P., Ravandi-Kashani F., Miranda R., Vadhan-Raj S., Koeppen H., Bueso-Ramos C.E. (2010). Acute erythroid leukemia as defined in the World Health Organization classification is a rare and pathogenetically heterogeneous disease. Mod. Pathol..

[5] Selby D.M., Valdez R., Schnitzer B., Ross C.W., Finn W.G. (2003). Diagnostic criteria for acute erythroleukemia. Blood.

[6] Arber D.A., Orazi A., Hasserjian R., Thiele J., Borowitz M.J., Le Beau M.M., Bloomfield C.D., Cazzola M., Vardiman J.W. (2016). The 2016 revision to the World Health Organization classification of myeloid neoplasms and acute leukemia. Blood.

[7] Arenillas L., Calvo X., Luno E., Senent L., Alonso E., Ramos F., Ardanaz M.T., Pedro C., Tormo M., Marco V. (2016). Considering Bone Marrow Blasts From Nonerythroid Cellularity Improves the Prognostic Evaluation of Myelodysplastic Syndromes. J. Clin. Oncol..

[8] Calvo X., Arenillas L., Luno E., Senent L., Arnan M., Ramos F., Ardanaz M.T., Pedro C., Tormo M., Montoro J. (2016). Erythroleukemia shares biological features and outcome with myelodysplastic syndromes with excess blasts: A rationale for its inclusion into future classifications of myelodysplastic syndromes. Mod. Pathol..

[9] Jogai S., Varma N., Garewal G., Das R., Varma S. (2001). Acute erythroleukemia (AML-M6)—A study of clinicohematological, morphological and dysplastic features in 10 cases. Indian J. Cancer.

[10] Olopade O.I., Thangavelu M., Larson R.A., Mick R., Kowal-Vern A., Schumacher H.R., Le Beau M.M., Vardiman J.W., Rowley J.D. (1992). Clinical, morphologic, and cytogenetic characteristics of 26 patients with acute erythroblastic leukemia. Blood.

[11] Domingo-Claros A., Larriba I., Rozman M., Irriguible D., Vallespi T., Aventin A., Ayats R., Milla F., Sole F., Florensa L. (2002). Acute erythroid neoplastic proliferations. A biological study based on 62 patients. Haematologica.

[12] Lessard M., Struski S., Leymarie V., Flandrin G., Lafage-Pochitaloff M., Mozziconacci M.J., Talmant P., Bastard C., Charrin C., Baranger L. (2005). Cytogenetic study of 75 erythroleukemias. Cancer Genet. Cytogenet..

[13] Mazzella F.M., Kowal-Vern A., Shrit M.A., Rector J.T., Cotelingam J.D., Schumacher H.R. (2000). Effects of multidrug resistance gene expression in acute erythroleukemia. Mod. Pathol..

[14] Grossmann V., Bacher U., Haferlach C., Schnittger S., Potzinger F., Weissmann S., Roller A., Eder C., Fasan A., Zenger M. (2013). Acute erythroid leukemia (AEL) can be separated into distinct prognostic subsets based on cytogenetic and molecular genetic characteristics. Leukemia.

[15] Atkinson J., Hrisinko M.A., Weil S.C. (1992). Erythroleukemia: A review of 15 cases meeting 1985 FAB criteria and survey of the literature. Blood Rev..

[16] Micci F., Thorsen J., Panagopoulos I., Nyquist K.B., Zeller B., Tierens A., Heim S. (2013). High-throughput sequencing identifies an NFIA/CBFA2T3 fusion gene in acute erythroid leukemia with t(1;16)(p31;q24). Leukemia.

[17] Srinivas U., Kumar R., Pati H.P., Saxena R., Tyagi S. (2007). Sub classification and clinico-hematological correlation of 40 cases of acute erythroleukemia—Can proerythroblast/myeloblast and proerythroblast/total erythroid cell ratios help subclassify?. Hematology.

[18] Mazzella F.M., Kowal-Vern A., Shrit M.A., Wibowo A.L., Rector J.T., Cotelingam J.D., Collier J., Mikhael A., Cualing H., Schumacher H.R. (1998). Acute erythroleukemia: Evaluation of 48 cases with reference to classification, cell proliferation, cytogenetics, and prognosis. Am. J. Clin. Pathol..

[19] Kowal-Vern A., Mazzella F.M., Cotelingam J.D., Shrit M.A., Rector J.T., Schumacher H.R. (2000). Diagnosis and characterization of acute erythroleukemia subsets by determining the percentages of myeloblasts and proerythroblasts in 69 cases. Am. J. Hematol..

[20] Santos F.P., Faderl S., Garcia-Manero G., Koller C., Beran M., O‘Brien S., Pierce S., Freireich E.J., Huang X., Borthakur G. (2009). Adult acute erythroleukemia: An analysis of 91 patients treated at a single institution. Leukemia.

[21] Pierdomenico F., Almeida A. (2013). Treatment of acute erythroleukemia with Azacitidine: A case series. Leuk. Res. Rep..

[22] Colita A., Belhabri A., Chelghoum Y., Charrin C., Fiere D., Thomas X. (2001). Prognostic factors and treatment effects on survival in acute myeloid leukemia of M6 subtype: A retrospective study of 54 cases. Ann. Oncol..

[23] Fenaux P., Mufti G.J., Hellstrom-Lindberg E., Santini V., Finelli C., Giagounidis A., Schoch R., Gattermann N., Sanz G., List A. (2009). Efficacy of azacitidine compared with that of conventional care regimens in the treatment of higher-risk myelodysplastic syndromes: A randomised, open-label, phase III study. Lancet Oncol..

[24] Garcia-Manero G., Jabbour E., Borthakur G., Faderl S., Estrov Z., Yang H., Maddipoti S., Godley L.A., Gabrail N., Berdeja J.G. (2013). Randomized open-label phase II study of decitabine in patients with low- or intermediate-risk myelodysplastic syndromes. J. Clin. Oncol..

[25] Pleyer L., Germing U., Sperr W.R., Linkesch W., Burgstaller S., Stauder R., Girschikofsky M., Schreder M., Pfeilstocker M., Lang A. (2014). Azacitidine in CMML: Matched-pair analyses of daily-life patients reveal modest effects on clinical course and survival. Leuk. Res..

[26] Thorpe M., Montalvao A., Pierdomenico F., Moita F., Almeida A. (2012). Treatment of chronic myelomonocytic leukemia with 5-Azacitidine: A case series and literature review. Leuk. Res..

[27] Fianchi L., Criscuolo M., Breccia M., Maurillo L., Salvi F., Musto P., Mansueto G., Gaidano G., Finelli C., Aloe-Spiriti A. (2013). High rate of remissions in chronic myelomonocytic leukemia treated with 5-azacytidine: Results of an Italian retrospective study. Leuk. Lymphoma.

[28] Fenaux P., Mufti G.J., Hellstrom-Lindberg E., Santini V., Gattermann N., Germing U., Sanz G., List A.F., Gore S., Seymour J.F. (2010). Azacitidine prolongs overall survival compared with conventional care regimens in elderly patients with low bone marrow blast count acute myeloid leukemia. J. Clin. Oncol..

[29] Dombret H., Seymour J.F., Butrym A., Wierzbowska A., Selleslag D., Jang J.H., Kumar R., Cavenagh J., Schuh A.C., Candoni A. (2015). International phase 3 study of azacitidine vs. conventional care regimens in older patients with newly diagnosed AML with >30% blasts. Blood.

[30] Kantarjian H.M., Thomas X.G., Dmoszynska A., Wierzbowska A., Mazur G., Mayer J., Gau J.P., Chou W.C., Buckstein R., Cermak J. (2012). Multicenter, randomized, open-label, phase III trial of decitabine versus patient choice, with physician advice, of either supportive care or low-dose cytarabine for the treatment of older patients with newly diagnosed acute myeloid leukemia. J. Clin. Oncol..

[31] Thepot S., Itzykson R., Seegers V., Recher C., Raffoux E., Quesnel B., Delaunay J., Cluzeau T., Marfaing Koka A., Stamatoullas A. (2014). Azacitidine in untreated acute myeloid leukemia: A report on 149 patients. Am. J. Hematol..

[32] Pleyer L., Burgstaller S., Girschikofsky M., Linkesch W., Stauder R., Pfeilstocker M., Schreder M., Tinchon C., Sliwa T., Lang A. (2014). Azacitidine in 302 patients with WHO-defined acute myeloid leukemia: Results from the Austrian Azacitidine Registry of the AGMT-Study Group. Ann. Hematol..

[33] Falantes J., Thepot S., Pleyer L., Maurillo L., Martínez-Robles V., Itzykson R., Bargay J., Stauder R., Venditti A., Martínez M.P., Seegers V. (2015). Azacitidine in older patients with acute myeloid leukemia (AML). Results from the expanded international E-ALMA series (E-ALMA+) according to the MRC risk index score. Blood.

[34] Pleyer L., Stauder R., Burgstaller S., Schreder M., Tinchon C., Pfeilstocker M., Steinkirchner S., Melchardt T., Mitrovic M., Girschikofsky M. (2013). Azacitidine in patients with WHO-defined AML—Results of 155 patients from the Austrian Azacitidine Registry of the AGMT-Study Group. J. Hematol. Oncol..

[35] Maurillo L., Venditti A., Spagnoli A., Gaidano G., Ferrero D., Oliva E., Lunghi M., D’Arco A.M., Levis A., Pastore D. (2012). Azacitidine for the treatment of patients with acute myeloid leukemia: Report of 82 patients enrolled in an Italian Compassionate Program. Cancer.

[36] Pleyer L., Burgstaller S., Stauder R., Girschikofsky M., Sill H., Schlick K., Thaler J., Halter B., Machherndl-Spandl S., Zebisch A. (2016). Azacitidine front-line in 339 patients with myelodysplastic syndromes and acute myeloid leukaemia: Comparison of French-American-British and World Health Organization classifications. J. Hematol. Oncol..

[37] Kadia T.M., Thomas X.G., Dmoszynska A., Wierzbowska A., Minden M., Arthur C., Delaunay J., Ravandi F., Kantarjian H. (2015). Decitabine improves outcomes in older patients with acute myeloid leukemia and higher blast counts. Am. J. Hematol..

[38] Mayer J., Arthur C., Delaunay J., Mazur G., Thomas X.G., Wierzbowska A., Ravandi F., Berrak E., Jones M., Li Y. (2014). Multivariate and subgroup analyses of a randomized, multinational, phase 3 trial of decitabine vs. treatment choice of supportive care or cytarabine in older patients with newly diagnosed acute myeloid leukemia and poor- or intermediate-risk cytogenetics. BMC Cancer.

[39] Gupta N., Miller A., Gandhi S., Ford L.A., Vigil C.E., Griffiths E.A., Thompson J.E., Wetzler M., Wang E.S. (2015). Comparison of epigenetic versus standard induction chemotherapy for newly diagnosed acute myeloid leukemia patients ≥60 years old. Am. J. Hematol..

[40] Lao Z., Yiu R., Wong G.C., Ho A. (2015). Treatment of elderly patients with acute myeloid leukemia with azacitidine results in fewer hospitalization days and infective complications but similar survival compared with intensive chemotherapy. Asia Pac. J. Clin. Oncol..

[41] Van der Helm L.H., Scheepers E.R., Veeger N.J., Daenen S.M., Mulder A.B., van den Berg E., Vellenga E., Huls G. (2013). Azacitidine might be beneficial in a subgroup of older AML patients compared to intensive chemotherapy: A single centre retrospective study of 227 consecutive patients. J. Hematol. Oncol..

[42] Jabbour E., Mathisen M.S., Garcia-Manero G., Champlin R., Popat U., Khouri I., Giralt S., Kadia T., Chen J., Pierce S. (2013). Allogeneic hematopoietic stem cell transplantation versus hypomethylating agents in patients with myelodysplastic syndrome: A retrospective case-control study. Am. J. Hematol..

[43] Ravandi F., Issa J.P., Garcia-Manero G., O’Brien S., Pierce S., Shan J., Borthakur G., Verstovsek S., Faderl S., Cortes J. (2009). Superior outcome with hypomethylating therapy in patients with acute myeloid leukemia and high-risk myelodysplastic syndrome and chromosome 5 and 7 abnormalities. Cancer.

[44] Almeida A., Ferreira A.R., Costa M.J., Silva S., Alnajjar K., Bogalho I., Pierdomenico F., Esteves S., Alpoim M., Braz G. (2017). Clinical outcomes of AML patients treated with Azacitidine in Portugal: A retrospective multicenter study. Leuk. Res. Rep..

[45] Hansen S.B., Dufva I.H., Kjeldsen L. (2012). Durable complete remission after azacitidine treatment in a patient with erythroleukaemia. Eur. J. Haematol..

[46] Hangai S., Nakamura F., Kamikubo Y., Honda A., Arai S., Nakagawa M., Ichikawa M., Kurokawa M. (2013). Erythroleukemia showing early erythroid and cytogenetic responses to azacitidine therapy. Ann. Hematol..

[47] Vigil C.E., Cortes J., Kantarjian H., Garcia-Manero G., Lancet J., List A. (2009). Hypomethylating Therapy for the Treatment of Acute Erythroleukemia Patients. Blood.

[48] King R.J., Crouch A., Radojcic V., Marini B.L., Perissinotti A.J., Bixby D. (2016). Therapeutic Outcomes of Patients with Acute Erythroid Leukemia Treated with Hypomethylating Agents. Blood.

[49] Uchida T., Hagihara M., Hua J., Inoue M. (2016). The effects of azacitidine on the response and prognosis of myelodysplastic syndrome and acute myeloid leukemia involving a bone marrow erythroblast frequency of >50. Leuk. Res..

[50] Steger G.G., Dittrich C., Chott A., Derfler K., Schwarzmeier J.D. (1989). Long-term remission in a patient with erythroleukemia following interferon-α treatment. J. Biol. Response Modif..

[51] Camera A., Volpicelli M., Villa M.R., Risitano A.M., Rossi M., Rotoli B. (2002). Complete remission induced by high dose erythropoietin and granulocyte colony stimulating factor in acute erythroleukemia (AML-M6 with maturation). Haematologica.

[52] Creusot F., Acs G., Christman J.K. (1982). Inhibition of DNA methyltransferase and induction of Friend erythroleukemia cell differentiation by 5-azacytidine and 5-aza-2’-deoxycytidine. J. Biol. Chem..

[53] Gambari R., del Senno L., Barbieri R., Viola L., Tripodi M., Raschella G., Fantoni A. (1984). Human leukemia K-562 cells: Induction of erythroid differentiation by 5-azacytidine. Cell Differ..

[54] Zucker R.M., Decal D.L., Whittington K.B. (1983). 5-Azacytidine increases the synthesis of embryonic hemoglobin (E2) in murine erythroleukemic cells. FEBS Lett..

[55] Ando T., Nishimura M., Oka Y. (2000). Decitabine (5-Aza-2′-deoxycytidine) decreased DNA methylation and expression of *MDR-1* gene in K562/ADM cells. Leukemia.

[56] Efferth T., Futscher B.W., Osieka R. (2001). 5-Azacytidine modulates the response of sensitive and multidrug-resistant K562 leukemic cells to cytostatic drugs. Blood Cells Mol. Dis..

[57] Pleyer L., Greil R. (2015). Digging deep into “dirty” drugs—Modulation of the methylation machinery. Drug Metab. Rev..

[58] Damaj G., Duhamel A., Robin M., Beguin Y., Michallet M., Mohty M., Vigouroux S., Bories P., Garnier A., El Cheikh J. (2012). Impact of azacitidine before allogeneic stem-cell transplantation for myelodysplastic syndromes: A study by the Societe Francaise de Greffe de Moelle et de Therapie-Cellulaire and the Groupe-Francophone des Myelodysplasies. J. Clin. Oncol..

[59] Figueroa M.E., Skrabanek L., Li Y., Jiemjit A., Fandy T.E., Paietta E., Fernandez H., Tallman M.S., Greally J.M., Carraway H. (2009). MDS and secondary AML display unique patterns and abundance of aberrant DNA methylation. Blood.

[60] Papaemmanuil E., Gerstung M., Bullinger L., Gaidzik V.I., Paschka P., Roberts N.D., Potter N.E., Heuser M., Thol F., Bolli N. (2016). Genomic Classification and Prognosis in Acute Myeloid Leukemia. N. Engl. J. Med..

[61] Dohner H., Estey E.H., Amadori S., Appelbaum F.R., Buchner T., Burnett A.K., Dombret H., Fenaux P., Grimwade D., Larson R.A. (2010). Diagnosis and management of acute myeloid leukemia in adults: Recommendations from an international expert panel, on behalf of the European LeukemiaNet. Blood.

[62] Cheson B.D., Greenberg P.L., Bennett J.M., Lowenberg B., Wijermans P.W., Nimer S.D., Pinto A., Beran M., de Witte T.M., Stone R.M. (2006). Clinical application and proposal for modification of the International Working Group (IWG) response criteria in myelodysplasia. Blood.

[63] R: The R Project for Statistical Computing. https://www.r-project.org/.

