# Supplementary material for: Clinical Outcomes of 217 Patients with Acute Erythroleukemia According to Treatment Type and Line: A Retrospective Multinational Study
Source: Int J Mol Sci. 2017 Apr 14;18(4):837. doi: 10.3390/ijms18040837 (PMC5412421; doi:10.3390/ijms18040837)
Supplement: Supplementary file 1 [file ijms-18-00837-s001.pdf]

Supplementary File

Table S1. Observed progression-free survival.

	n Patients	*n* Events	Median PFS, mo (95% CI)	One-Year PFS, % (95% CI)
Total treated cohort [1]	177	139	7.1 (6.3-9.4)	35.6 (28.7-44.0)
HMA all lines [2]	74	65	5.1 (3.4-9.2)	26.9 (18.2-39.6)
HMA ≥2nd line [3]	33	31	3.4 (2.0-6.3)	12.6% (5.0-31.5)
HMA 1st line [4]	39	32	9.4 (4.2-14.5)	40.6 (27.3-60.2)
ICT 1st line [5]	103	74	8.0 (6.9-14.5)	41.8 (32.5-53.7)

[1] Includes HMA 1st line, HMA ≥ 2nd line, and ICT 1st line;
[2] Data on PFS was not available for n = 14 patients;
[3] Data on PFS was not available for n = 12 patients;
[4] Data on PFS was not available for n = 2 patients;
[5] Data on PFS was not available for n = 19 patients.

Table S2. Observed overall survival in the overall sample and in treatment subgroups.

	n Patients	*n* Events	Median OS, mo (95% CI)	One-Year Survival, % (95% CI)
Total treated cohort [1]	205	142	11.1 (9.8-14.3)	49.1 (42.2-57.2)
HMA all lines [2]	84	58	12.3 (9.8-14.3)	52.5 (42.1-65.5)
HMA ≥2nd line [3,*]	41	28	9.8 (4.6-13.5)	38.0 (24.2-59.6)
HMA 1st line [4,*]	41	29	13.7 (12.3-20.5)	65.8 (52.2-82.9)
ICT 1st line [5]	121	84	10.5 (9.1-20.0)	46.7 (37.9-57.5)

[1] Includes HMA 1st line, HMA ≥ 2nd line, and ICT 1st line;
[2] Data on OS was not available for n = 4 patients;
[3] Data on OS was not available for n = 1 patient;
[4] Data on OS was not available for n = 1 patient;
[5] Data on OS was not available for n = 1 patient;
* Data on line of treatment was not available for n = 2 patients.

Table S3. Distribution of causes of death by treatment group (censored at BMT).

Cause of Death	HMA (n=84 [1])	ICT (3+7) (n=121 [2])	p-Value (Chi-Square-Test)
AML progression, n/n (%)	31/58 (53)	55/84 (65)	0.269
Infection, n/n (%)	10/58 (17)	11/84 (13)	0.465
Others, n/n (%)	6/58 (10)	6/84 (7)	0.467
Unknown, n/n (%)	11/58 (19)	12/84 (14)	0.384
Total deaths, n/n (%)	58/84 (69)	84/121 (69)	1

[1] Survival Data was not available for n = 4 patients. [2] Survival Data was not available for n = 1 patient.
